# Non-ionizing Imaging for the Emergency Department Assessment of Pediatric Minor Head Trauma

**DOI:** 10.3389/fped.2022.881461

**Published:** 2022-05-11

**Authors:** Alessia Cicogna, Giulia Minca, Francesca Posocco, Federica Corno, Cecilia Basile, Liviana Da Dalt, Silvia Bressan

**Affiliations:** Division of Pediatric Emergency Medicine, Department of Women’s and Children’s Health, University of Padova, Padua, Italy

**Keywords:** pediatric minor head trauma, traumatic brain injury, pediatric, skull ultrasound, near-infrared spectroscopy, magnetic resonance imaging

## Abstract

Minor blunt head trauma (MHT) represents a common reason for presentation to the pediatric emergency department (ED). Despite the low incidence of clinically important traumatic brain injuries (ciTBIs) following MHT, many children undergo computed tomography (CT), exposing them to the risk associated with ionizing radiation. The clinical predictions rules developed by the Pediatric Emergency Care Applied Research Network (PECARN) for MHT are validated accurate tools to support decision-making about neuroimaging for these children to safely reduce CT scans. However, a few non-ionizing imaging modalities have the potential to contribute to further decrease CT use. This narrative review provides an overview of the evidence on the available non-ionizing imaging modalities that could be used in the management of children with MHT, including point of care ultrasound (POCUS) of the skull, near-infrared spectroscopy (NIRS) technology and rapid magnetic resonance imaging (MRI). Skull ultrasound has proven an accurate bedside tool to identify the presence and characteristics of skull fractures. Portable handheld NIRS devices seem to be accurate screening tools to identify intracranial hematomas also in pediatric MHT, in selected scenarios. Both imaging modalities may have a role as adjuncts to the PECARN rule to help refine clinicians’ decision making for children at high or intermediate PECARN risk of ciTBI. Lastly, rapid MRI is emerging as a feasible and accurate alternative to CT scan both in the ED setting and when repeat imaging is needed. Advantages and downsides of each modality are discussed in detail in the review.

## Introduction

Minor blunt head trauma (MHT), remains one of the most common reasons for children to present to the Emergency Department (ED) in high income countries (1, 2). MHT infrequently results in fractures of the skull and/or traumatic brain injuries (TBIs), and only approximately 1% of children, overall, will sustain clinically important TBIs (ciTBIs). These include any of the following: death, neurosurgery, intubation for >24, or hospitalization for 2 or more nights in association with TBI on computed tomography (CT) (3, 4). While CT of the head is the gold standard to diagnose skull fractures and TBIs, it exposes patients to ionizing radiation. Its use is associated with an increased lifetime risk of malignancy, especially in children, although the most recent CT technology and the optimization of CT radiation dose mitigate this risk (5–9).

**FIGURE 1:**
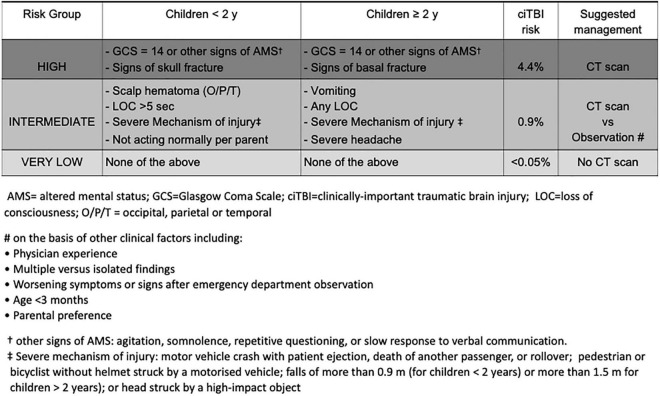
Pediatric Emergency Care Applied Research Network algorithms for the emergency department management of minor head trauma – Adapted from (3).

Over the past two decades many clinical prediction rules, including symptoms and signs from history and physical examination, have been developed to support clinical decision making on CT scan, in order to optimize its use and reduce unnecessary radiation exposure (3, 4, 10, 11). Of these, the two age-specific prediction rules derived and validated by the Pediatric Emergency Care Applied Research Network (PECARN) (3) and externally validated in several studies (4, 12, 13) showed to be methodologically robust and highly accurate in identifying children at low risk of ciTBIs, following a minor head trauma defined by a Glasgow Coma Scale (GCS) score of 14 or 15 on assessment (Figure 1). Of note, these rules do not apply to children with suspected abusive head trauma. For children with accidental MHT, in the absence of the rule predictor variables a CT scan can be safely avoided. In the presence of the PECARN rule predictor variables, the risk of ciTBI differs according to the type and number of predictors. The two age-specific PECARN rules, one for pre-verbal children <2 years, and one for those ≥2 years, include six predictor variables. Of each age-specific rule predictors, four are associated with an intermediate risk of ciTBI. Based on the PECARN rule risk stratification algorithm, in the presence of any of the four intermediate-risk predictors, clinicians can choose to observe the patient in the ED for a period of time, or to immediately obtain a CT. For children at intermediate risk of ciTBI clinicians may favor CT over observation, based on the presence of multiple versus isolated findings, including physician experience, worsening symptoms or signs during observation, age <3 months, and parental preference, among other factors. Two of the six PECARN rule predictor variables were found to be associated with a higher risk of ciTBI. For children at high risk of ciTBI based on the PECARN rule, the trade-off between the risk of missing a ciTBI and the risk associated with CT-related radiation exposure is in favour of obtaining a CT scan.

The PECARN rules have been widely implemented worldwide, often with some adaptations, and are routinely used to assist clinical practice in several EDs (14–18). Since their publication, several secondary analyses of the PECARN rules parent study (3) and of their largest external validation study (4) contributed to best define the risk of ciTBI for children presenting with one of the rule predictor variables in isolation or associated with other predictors (19–27). These data provide clinicians with additional information to further refine their decision-making based on a more precise risk stratification for ciTBI within specific subgroups of patients. Although the use of the PECARN rules has led to significant decrease in CT scans in many settings (28–30), unnecessary CT scans are still often performed (31).

The ability to further refine the risk of ciTBI through the use of bedside non-ionizing imaging modalities represents an opportunity to further reduce unnecessary CT scans, their related radiation exposure, the possible need for sedation in uncooperative children and the associated costs. With this respect, point of care ultrasound (POCUS) of the skull and bedside near-infrared spectroscopy technology (NIRS) devices have the potential to be used as adjuncts to the PECARN rules to further contribute to patient risk refinement and selection for neuroimaging. Another opportunity to reduce radiation exposure is the use of magnetic resonance imaging (MRI) in place of CT scan for the diagnosis of TBIs. This imaging modality, however, traditionally requires that the child remains motionless for several minutes and usually needs sedation, making MRI not suitable or impractical in the setting of acute head trauma. More recently, rapid or fast MRI motion-tolerant abbreviated sequence protocols, that have been used to reduce radiation exposure in children with shunt treated hydrocephalus for more than a decade (32), have shown promise as a feasible and accurate alternative to CT in clinically stable children with concern for TBI.

In this narrative review we will summarize the evidence on the available non-ionizing imaging modalities, including skull POCUS, NIRS bedside devices and rapid MRI, in the management of children with accidental MHT.

Although this was not a systematic review we are providing herein the details of the literature search used to identify the key evidence presented in our review. We searched PubMed focusing on three main concepts: (1) head trauma; (2) specific non-ionizing imaging techniques (i.e., skull ultrasound, transfontanelle ultrasound; near infrared spectroscopy brain scanner; and rapid magnetic resonance imaging); and (3) the pediatric field. We used the following search terms for each of the above concepts: (1) “head trauma” OR “head injury” OR “traumatic brain injury”; (2) “ultrasound” OR “ultrasonography”; “near-infrared spectroscopy”; (“quick brain” OR “rapid sequence” OR “fast”) AND (MRI OR magnetic resonance imaging); (3) child* OR pediatric* OR paediatric*. We excluded articles that focused on moderate or severe head injury, on non-accidental head trauma and non-English articles. We also checked the reference lists of selected papers to screen for further possibly relevant articles.

## Point of Care Ultrasound

Point of care ultrasound (POCUS) (33) is widely used in EDs worldwide (34) as it is rapid, non-invasive, inexpensive, and it does not expose children to ionizing radiation. Its role is well established for the assessment of soft tissues and parenchymatous organs or for determining the presence of fluid collections, but it is also being increasingly used to study bone lesions. With this respect, it has been demonstrated that clinicians with adequate training can use POCUS to accurately diagnose bone fractures, both in children and adults (35).

In the context of pediatric MHT, this technique is particularly useful when a scalp hematoma is present and clinical signs of a palpable skull fracture may be unclear or doubtful to define the presence of an underlying depressed or complicated fracture of the skull (36, 37). Two recent meta-analyses (38, 39) evaluated the accuracy of skull POCUS performed by ED physicians in identifying skull fractures in children with head trauma. Both included prospective studies that compared POCUS results with CT scan findings (reference standard). Both meta-analyses, although slightly different in the methods and in the number of studies included [six studies in Gordon et al. (39) and seven in Alexandridis et al. (38)] found a pooled sensitivity of 91% and a pooled specificity of 96%, confirming the accuracy of POCUS in detecting skull fractures. Overall, the largest meta-analysis analyzed 925 patients, with study samples ranging from 21 to 538 patients and percentage of skull fractures on CT ranging from 10 to 77%.

One of the limitations of US is the training required to achieve the ability to accurately identify disease, which makes this imaging modality operator-dependent. However, limited training is required to accurately identify skull fractures (38). A recent multicentre study showed that clinicians who were mostly novices to skull POCUS, were able to correctly classify the type of skull fracture as linear, depressed, or complex in 84.4% of cases as compared with CT scan results (k statistic of 0.75, 95% CI 0.70–0.84) (40). Participating clinicians were able to achieve such good results after receiving 2 video didactic training sessions in skull POCUS techniques, and hands-on training done locally at each site, which included, at some sites, homemade, low-cost ultrasound phantoms for instruction and practice (41). The clinicians performing the POCUS examinations were trained to look for cortical skull irregularities visible in multiple orientations to be considered a true positive fracture. They also had to demonstrate 10 successful skull POCUS examinations on patients younger than 2 years under the supervision of the site POCUS lead.

While skull POCUS should not be used as a screening tool for intracranial injuries (15, 18), based on the identification of a skull fracture *per se*, it could help better select patients warranting a head CT by direct visualization of fracture characteristics (18). As a matter of fact, differently from skull x-rays, POCUS can better define whether a fracture is depressed, diastatic or comminuted. The PECARN rule predictor “signs of palpable skull fractures” for younger children is associated with a high-risk of ciTBI, suggesting that a CT scan should be obtained in these patients. Fractures which can be palpated on physical examination (due to a gap or step-off in the fracture margins) are more often associated with ciTBIs, and depressed fractures can sometimes require surgery *per se*, depending on the depth of depression (3). The clinical finding of a “palpable skull fracture,” however, was previously reported to have a low interobserver agreement among clinicians (kappa index of 0.67 with a lower 95% confidence interval limit of 0.41) (42). Skull POCUS, by defining the presence and, most importantly, characteristics of a skull fracture, appears to be a useful adjunctive tool to refine decision-making on CT scan in young children with “signs of palpable skull fractures,” especially when this clinical finding may be unclear or doubtful.

In addition to POCUS of the skull, some authors have proposed transfontanelle US, in infants younger than 1 year of age, as a useful tool to assess for intracranial lesions after MHT (43). One important limitation, however, is its inability to accurately identify extra-axial hematomas. For this reason, the Italian and Australasian guidelines recommend not to routinely use transfontanelle US for diagnosing intracranial injuries, especially prior to, or in lieu of, a head CT (13, 42).

Last, although transcranial doppler is being increasingly used to aid in the diagnosis and monitoring of intracranial hypertension following moderate or severe pediatric traumatic brain injuries, its role in the acute ED management of children with MHT is yet to be defined (44–46).

## Near Infrared Spectroscopy

Near-infrared spectroscopy (NIRS) is a non-invasive, affordable, easy-to-learn and to-use, non-radiating technology, which can detect the presence of intracranial hematomas following a head trauma. A NIRS apparatus consists of a near-infrared light source that shines light (within a wavelength of 700–950 nm) to the head, and a detector that receives the light after it has interacted with the tissues. Under normal circumstances, the brain’s absorption is symmetrical.

The detection of intracranial hematomas is based on the differential near-infrared light absorption of extravascular hemoglobin within the injured side of the brain compared to the uninjured brain (47). Hand-held portable NIRS devices allow for examination of head trauma patients at the bedside, and can be used in both the pre-hospital and in-hospital setting. The handheld brain scanner is placed successively in the left and right frontal, temporal, parietal, and occipital regions of the head. The device electronically calculates the difference in optical density (ΔOD) between the right and left side in each of the four regions on a pairwise basis. The formula used for this purpose is ΔOD = log10 (IN/IH), where IN is the intensity of the reflected light on the presumed normal side, and IH is the intensity of the reflected light on the presumed abnormal side (47). A positive test result is defined by a ΔOD > 0.2 between two symmetric regions, based on a pilot study of patients with hematomas and healthy controls and set to maximize sensitivity and specificity (48).

Seven studies, all prospective, investigated the accuracy of this technology in detecting CT-diagnosed intracranial hemorrhages in children, six of which are included in a recent systematic review (47). Overall, the seven studies comprise a total of 657 pediatric patients undergoing both a NIRS assessment and a head CT (study samples ranging from 18 to 344) (49–54). All studies were conducted in the in-hospital setting (six in the ED and one in the intensive care unit) using different inclusion criteria. The rate of positive CT scans was highly variable across studies (between 4.7 and 42.9%). Sensitivities ranged between 58.3 and 100%, and specificities between 65.3 and 98.7%. Of note, in the largest and most recent multicenter study conducted in the United States using the latest brain scanner model, NIRS technology demonstrated a sensitivity of 58.3% (21/36) and specificity of 67.9% (209/308) for hematomas of any size and location. Considering only hematomas within the NIRS device detection limits, the sensitivity was 81% (13/16) and specificity 67.4% (221/328) (55). None of the intracranial hemorrhages missed by NIRS technology needed neurosurgery. The negative predictive values were consistently high across studies, between 98 and 100%, meaning that a negative result on the NIRS assessment is highly predictive of the absence of intracranial hemorrhages, within the device detection limits. The assessment with the NIRS brain scanner can be easily completed in approximately 90% of children, mostly within 5 min (54, 55). One of the hand-held NIRS devices has recently been cleared by the FDA for the detection of supratentorial hematomas also in patients aged 2 years and older (56).

Despite several advantages, NIRS technology bears some limitations that need to be acknowledged for a correct use and interpretation of its results. First, NIRS devices are able to detect only hematomas > 3.5 mL in volume and within a depth of 2.5 cm of the brain surface. Secondly, it is not accurate in the presence of bilateral hematomas, as the technology relies on the pairwise comparison of light absorption between the two hemispheres. Third, scalp hematomas may be confounding factors for NIRS technology, because blood contained within a scalp hematoma can alter the absorption of the NIR light and cause a false-positive result. Furthermore, NIRS technology is unable to precisely determine the location (eg, subdural vs epidural) and volume extent of intracranial hematomas, and it is only useful in the presence of acute hematomas (<12 h). In addition, it can be difficult to apply on thick hair or injured skin. Lastly, operators need to maintain proficiency in standardized and meticulous positioning of the device to avoid hair, foreign bodies, and scalp hematomas. Optimal pressure of the probe against the scalp also needs to be ensured to yield reliable measurements (55).

In summary, NIRS technology appears to be a useful adjunctive tool to the PECARN head trauma rule to refine decision-making on CT for patients at PECARN intermediate risk of ciTBI. The high negative predictive value of NIRS assessment may help reduce the number of CT scans. Further investigation, however, is warranted to determine its clinical impact and best use in practice.

## Rapid Magnetic Resonance Imaging

In 2002, “quick-brain” MRI was introduced as an alternative technique to CT scanning for the evaluation of children with hydrocephalus (32, 57). Since then, its use has been extended to several other conditions, including MHT. Fast MRI or Rapid-sequence MRI of the brain is a limited-sequence MRI protocol, which reduces the time to image acquisition. This allows the detection of TBIs during the acute assessment of MHT, while avoiding exposure to ionizing radiation from head CT scans. A recent systematic review, without meta-analysis, included 13 studies on the use of rapid-sequence MRI in children. Of these, seven included children with head trauma as a sole inclusion criterion, three included patients meeting various indications to undergo neuroimaging, and three focused exclusively on abusive head trauma (58). An additional relevant study was published concurrently to the systematic review (59). Of the eight studies considered for this review (59–66), samples ranged between 23 and 233 patients and only two were prospective (60, 61). The average reported time to imaging completion varied between 1 and 16 min, depending on the MRI protocols used (58). Rapid MRI was successfully completed in 99% of children with head trauma, as reported by the largest prospective study by Lindberg et al. where the median time to completion was 6 min (60). These data show that rapid MRI is a feasible option for stable children with MHT in the ED. With respect to its diagnostic accuracy, although difficult to compare across studies due to the variable rapid MRI protocols used, this technique resulted overall comparable to CT for the detection of TBIs (sensitivity between 85 and 100%, and specificity between 83 and 100%), while less sensitive for linear non-displaced skull fractures (with wide variability in sensitivity across studies, between 34 and 100%) (58, 59). The accuracy in detecting linear skull fractures was reported to remain limited, even when complemented by a black bone sequence (62), although missed fractures never required neurosurgical intervention (58, 59, 63, 67). CT scans and rapid-sequence MRIs, in the relevant studies (59–66) were mostly performed within a time interval of 48 h. It is important to note that the variable time interval between CT scans and rapid-sequence MRIs may have affected the sensitivity of the latter for minor intracranial hemorrhages. Rapid MRI is particularly recommended in the assessment of MHT patients with persistent neurologic symptoms despite normal CT findings or in lieu of repeat CT (66). While some authors suggest rapid MRI being most helpful as a follow up imaging in patients with known TBIs (65), others reported rapid MRI to be slightly superior to CT for the detection of specific TBIs such as extradural and subdural hematomas, parenchymal contusions and white matter axonal injuries, although without a statistically significant difference (59, 60, 64, 66). The sensitivity of rapid MRI seems to be dependent on the type of sequence protocol used (65, 68, 69), other than the medical expertise of the radiologist (60, 70). Based on the feasibility and accuracy data of rapid MRI, this imaging modality seems to fit the ED needs for a timely diagnosis and a rapid throughput. However, institutions should integrate rapid MRI into acute pediatric TBI management judiciously, relying on the clinical context and institutional capabilities, especially for the prognostic and legal implications (58). Unfortunately, the infrastructural accessibility of rapid MRI is limited compared to CT (71) and it requires experienced and qualified staff for proper exam reading and interpretation, in order to avoid pitfalls. In addition, its use should be based on pre-defined and established criteria, as its actual diagnostic accuracy in the broader population of children with head trauma remains unknown (72, 73). Until consensus is achieved on the best protocol to be used and procedures are standardized, this imaging modality should be reserved to settings with high volumes of head trauma patients, able to perform MRI rapidly and safely in children, with experienced and qualified staff for best imaging acquisition, reading and interpretation, in order to ensure MRI is equivalent to a head CT scan in terms of utility (15).

## Conclusion

POCUS of the skull, NIRS technology and rapid MRI bear great potential to reduce CT-related radiation exposure in children with MHT. Their advantages, limitations and potential role in the management of pediatric MHT are summarized and compared in Table 1. While the availability and accuracy of rapid MRI are setting dependent and NIRS brain scanners are still not widespread in the ED setting, POCUS is already routinely used in EDs worldwide. This facilitates its implementation in the approach to pediatric MHT. Although operator dependent, its ability to more objectively define the PECARN risk factor of “signs of palpable skull fracture” can substantially contribute to refine clinical decision making in patients with this clinical sign. When implementing non-ionizing imaging for the management of MHT in the ED, continuous monitoring of its impact on patient health outcomes and CT scan use is warranted.

**TABLE 1:** Advantages, limitations, and role in the management of pediatric mild head trauma of non-radiating imaging techniques.

	Advantages	Limitations	Role in the management of pediatric MHT
POCUS of the skull	• Affordable • Bedside technology • Rapid learning • No need for sedation • Detection and characterization of skull fractures	• Inadequate for identifying intracranial injuries • Operator dependent	Adjunctive tool to PECARN head trauma prediction rule to refine decision-making on CT for younger children with the high-risk predictor “signs of palpable skull fractures” by defining the actual presence and characteristics of underlying fractures of the skull
NIRS	• Affordable • Bedside technology • Rapid learning • No need for sedation • Detection of intracranial hemorrhages	• For acute bleeds only (<12 h) • Limited to intracranial hemorrhage depth/volume • Inaccurate for bilateral hemorrhages • Impaired detection with scalp hematomas, thick hair • Operator dependent	Potential adjunctive tool to the PECARN head trauma rule to refine decision-making on CT for patients at PECARN high or intermediate risk of ciTBI Further investigation is warranted to determine its clinical impact and best use in the ED
Rapid MRI	• No sedation (feasible without sedation also in young children) • Compatible with ED pace and flow • Accurate for TBI	• Limited accessibility • Costs • Less sensitive to linear skull fractures • Lack of consensus about best protocol to use	Feasible and accurate alternative to CT for stable children with MHT to detect ciTBI in the ED, as well as surveillance imaging in lieu of repeat CT Good option for repeat imaging depending on detail needed

*ciTBI, clinically important traumatic brain injury; CT, computed tomography; ED, emergency department; MHT, mild head trauma; MRI, magnetic resonance imaging; NIRS, near infrared spectroscopy; PECARN, Pediatric Emergency Care Applied Research Network; POCUS, point of care ultrasound; TBI, traumatic brain imaging.*

## Author Contributions

SB conceived the theme and structure of this review with input from LD. AC coordinated the residents working group, searched the literature, drafted the manuscript, and revised the final version. GM, FC, FP, and CB searched the literature and drafted the manuscript. SB contributed to drafting the manuscript and critically reviewed and revised its final version. LD critically revised the manuscript. All authors read and approved the submitted version.

## Conflict of Interest

The authors declare that the research was conducted in the absence of any commercial or financial relationships that could be construed as a potential conflict of interest.

## Publisher’s Note

All claims expressed in this article are solely those of the authors and do not necessarily represent those of their affiliated organizations, or those of the publisher, the editors and the reviewers. Any product that may be evaluated in this article, or claim that may be made by its manufacturer, is not guaranteed or endorsed by the publisher.

## Abbreviations

ciTBI: clinically important traumatic brain injury
CT: computed tomography
ED: emergency department
MHT: minor head trauma
MRI: magnetic resonance imaging
NIRS: near infrared spectroscopy
PECARN: Pediatric Emergency Care Applied Research Network
POCUS: point of care ultrasound
US: ultrasound.
